# Relationship between the Montreal Cognitive Assessment and Mini-mental State Examination for assessment of mild cognitive impairment in older adults

**DOI:** 10.1186/s12877-015-0103-3

**Published:** 2015-09-07

**Authors:** Paula T. Trzepacz, Helen Hochstetler, Shufang Wang, Brett Walker, Andrew J. Saykin, for the Alzheimer’s Disease Neuroimaging Initiative

**Affiliations:** Indiana University School of Medicine, Indianapolis, IN USA; Eli Lilly and Company or one of its subsidiaries, Lilly Corporate Center, Indianapolis, IN USA; University College of London, Gower Street, London, UK

## Abstract

**Background:**

The Montreal Cognitive Assessment (MoCA) was developed to enable earlier detection of mild cognitive impairment (MCI) relative to familiar multi-domain tests like the Mini-Mental State Exam (MMSE). Clinicians need to better understand the relationship between MoCA and MMSE scores.

**Methods:**

For this cross-sectional study, we analyzed 219 healthy control (HC), 299 MCI, and 100 Alzheimer’s disease (AD) dementia cases from the Alzheimer’s Disease Neuroimaging Initiative (ADNI)-GO/2 database to evaluate MMSE and MoCA score distributions and select MoCA values to capture early and late MCI cases. Stepwise variable selection in logistic regression evaluated relative value of four test domains for separating MCI from HC. Functional Activities Questionnaire (FAQ) was evaluated as a strategy to separate dementia from MCI. Equi-percentile equating produced a translation grid for MoCA against MMSE scores. Receiver Operating Characteristic (ROC) analyses evaluated lower cutoff scores for capturing the most MCI cases.

**Results:**

Most dementia cases scored abnormally, while MCI and HC score distributions overlapped on each test. Most MCI cases scored ≥17 on MoCA (96.3 %) and ≥24 on MMSE (98.3 %). The ceiling effect (28–30 points) for MCI and HC was less using MoCA (18.1 %) versus MMSE (71.4 %). MoCA and MMSE scores correlated most for dementia (*r* = 0.86; versus MCI *r* = 0.60; HC *r* = 0.43). Equi-percentile equating showed a MoCA score of 18 was equivalent to MMSE of 24. ROC analysis found MoCA ≥ 17 as the cutoff between MCI and dementia that emphasized high sensitivity (92.3 %) to capture MCI cases. The core and orientation domains in both tests best distinguished HC from MCI groups, whereas comprehension/executive function and attention/calculation were not helpful. Mean FAQ scores were significantly higher and a greater proportion had abnormal FAQ scores in dementia than MCI and HC.

**Conclusions:**

MoCA and MMSE were more similar for dementia cases, but MoCA distributes MCI cases across a broader score range with less ceiling effect. A cutoff of ≥17 on the MoCA may help capture early and late MCI cases; depending on the level of sensitivity desired, ≥18 or 19 could be used. Functional assessment can help exclude dementia cases. MoCA scores are translatable to the MMSE to facilitate comparison.

**Electronic supplementary material:**

The online version of this article (doi:10.1186/s12877-015-0103-3) contains supplementary material, which is available to authorized users.

## Background

Office-based, multi-domain cognitive tests are commonly administered in clinical situations to evaluate patients with cognitive impairment. Galvin and Sadowski recently wrote clinical recommendations for primary care physician evaluation of older patients for cognitive impairment, emphasizing the need to look for early warning signs where formal cognitive testing can aid detection [1]. Though there are a number of possible tests, they recommend the Mini-Mental State Examination (MMSE) [2], the most widely used cognitive screening test used by physicians for general cognitive evaluation, and also the newer Montreal Cognitive Assessment (MoCA) [3]. The MMSE is also commonly used as a proxy for staging of Alzheimer’s disease (AD) [4].

One problem with the MMSE is its ceiling effect or limited dynamic performance range for normal individuals, which increases the likelihood that persons in predementia stages score within the normal range (24 and above) [5, 6]. Its poor sensitivity for distinguishing mild cognitive impairment (MCI) is well-described and can be attributed to a lack of complexity as well as the absence of executive function items [7–10]. As research increasingly focuses on milder stages of AD [11], options other than the MMSE are needed for clinicians for earlier diagnosis and management.

Though it offers many of the same advantages of the MMSE, the MoCA was developed as a more challenging test that includes executive function, higher-level language, and complex visuospatial processing to enable detection of mild impairment with less ceiling effect [3]. Greater sensitivity to detect mild levels of cognitive impairment has been reported for the MoCA in MCI and AD dementia [3, 12–14], stroke and transient ischemic attack patients [15] and Parkinson’s disease [16]. Freitas et al. reported better longitudinal sensitivity for MoCA than MMSE [13]. Lam et al. found a higher correlation of the MoCA than MMSE with neuropsychological tests for memory, executive functioning, visuospatial, and the Mattis Dementia Rating Scale [17].

The initial MoCA validation study required an MMSE score of at least 17 for inclusion and used cutoff values of ≤25 on both tests to denote abnormal scores consistent with amnestic MCI (Peterson criteria) or mild AD [3]. Mean scores in HC, MCI and mild AD dementia groups were lower on the MoCA than MMSE, though the overall correlation between tests was high (*r* = 0.87). MoCA scores for MCI subjects were detected as abnormal in 73 % of those whose MMSE was normal. The MoCA had greater sensitivity than MMSE in detecting MCI versus HCs (90 % versus 18 %); however, specificity was lower for the MoCA than MMSE (87 % versus 100 %) [3].

Subsequent studies explored lower MoCA cutoff scores to increase specificity for detection of cognitive impairment as compared to HCs. Using the Peterson criteria for amnestic MCI, Luis et al. found that an MMSE cutoff ≤24 did not distinguish HCs from MCI; however, the MoCA, using a cutoff of ≤23, with 96 % sensitivity and 95 % specificity, did [12]. These investigators and others who have evaluated lower MoCA cutoff values of ≤23 [12, 18, 19] and ≤20 [20] report increased detection of impaired MCI cases from HCs but risk leaving more subtly impaired MCI cases within the normal range.

Thus, current MoCA literature has focused on MCI defined more consistent with what is now considered late MCI. Also, MCI and dementia often comprised a cognitively impaired group where identifying cutoff values from HCs were sought. As MCI becomes a more commonly targeted population for clinical trials, the ability to capture the full range of MCI cases is crucial. Further, for clinicians wishing to detect cognitive impairment earlier, knowing the MCI score range using an office-based test like the MoCA would be very helpful. More sensitive neuropsychological tests would need to be applied secondarily to separate those with cognitive complaints representing the earliest MCI cases from HCs [21, 22]. Because functional impairment is required for a dementia diagnosis, this could be a differentiating feature from MCI when cognitive scores overlap. Accordingly, the Alzheimer’s Disease Neuroimaging Initiative (ADNI) study has extended the range of their designation of MCI cases beyond the Peterson criteria to include those at earlier stages. These early MCI cases often perform in the normal range on routine office-based tests, such as the MoCA and MMSE, whereas late MCI cases can score in the mild dementia range [22].

Our research purpose differed from prior reports comparing the MoCA to the MMSE in that we wished to establish a MoCA score range that captures as many MCI cases as reasonable, including those at a very early stage, as designated using ADNI study criteria. We analyzed the relationship between MoCA and MMSE scores with their distributions, equivalent scores, cutoff values for MCI versus AD dementia, and contribution of domain subscores in differentiating MCI from HC groups using ADNI data.

## Methods

### Subjects and design

This is a cross-sectional study analyzing 618 cases from the ADNI-GO and ADNI-2 databases where both MMSE and MoCA tests were performed within +/− 90 days of the clinical diagnosis. Data were downloaded from the October 2012 release [http://adni.loni.ucla.edu/]. ADNI is a multi-site, multi-study program funded by a public and private partnership to investigate whether the combination of neuroimaging, biological markers, and clinical and neuropsychological assessments can accurately track progression in AD [23]. Data are publicly available to the scientific community for analyses. Informed consent is collected through the participating ADNI sites. The ADNI-GO and ADNI-2 studies were conducted according to Good Clinical Practice guidelines, US 21CFR Part 50 – Protection of Human Subjects, and Part 56 – Institutional Review Boards (IRBs)/Research Ethics Boards (REBs), and pursuant to state and federal HIPAA regulations. Study protocols were approved by each site’s IRB/REB (Additonal file 1). For up-to-date information, see www.adni-info.org.

At the entry visit into ADNI, cohort subjects received an initial diagnosis according to certain definitions. AD dementia subjects had MMSE scores between 20–26 (inclusive), Clinical Dementia Rating Scale (CDR) scores of either 0.5 or 1.0, and all met National Institute of Neurological and Communicative Disorders and Stroke/Alzheimer’s Disease and Related Disorders Association criteria for probable AD. MCI subjects had a memory complaint, and MMSE scores between 24–30, objective memory loss as measured by education-adjusted scores on the Wechsler Memory Scale (WMS) Logical Memory II, CDR score of 0.5, absence of significant levels of impairment in other cognitive domains, essentially preserved activities of daily living, and an absence of dementia. In subsequent visits, diagnoses could change and were not restricted by these entry score ranges on the MMSE. For our study, follow-up visits (minimum of 6 months from entry) were used for the MMSE/MoCA study visit in all except three subjects.

We studied 618 cases from 219 cognitively normal HC, 299 MCI, and 100 AD dementia cases. Early and late MCI designations were not distinguished for this analysis. If there were multiple test measurements within the 90-day time range, the values closest in time to clinical diagnosis were selected.

### Procedures

#### Tests

The MMSE [2] is a widely used cognitive screening test, where scores from 24 to 30 are considered within the normal range. Items address orientation, memory, recall, attention, naming objects, following verbal and written commands, writing a sentence, and copying a figure.

The MoCA [3] was developed more recently to address the shortcomings of the MMSE in detecting MCI. Its score range is the same as the MMSE (0–30), but has additional, more complex tasks including executive function. Items address orientation, drawing figures, processing speed, naming objects, memory, recall, attention, vigilance, repetition, verbal fluency, and abstraction. The MoCA adds one point for those whose educational level is 12 or fewer years.

#### Strategy to address MCI overlap scores

We anticipated overlap of the MCI MoCA score range with the other diagnoses. Therefore, we evaluated whether other methods could help distinguish HC and AD dementia cases that score in the MCI range on the MoCA using a functional scale and MMSE and MoCA domain subscores.

The Functional Activities Questionnaire (FAQ) [24] measures instrumental activities of daily living (IADLs), such as preparing balanced meals and managing personal finances. The care partner is interviewed about the patient’s ability to carry out ten activities. Each activity is rated on a scale from 0 (normal) to 3 (dependent) with a maximum total score of 30. Scoring 3 points on at least 3 activities is the recommended cutoff to indicate impaired function consistent with dementia [24].

Domains of items (≥4 points per domain) within the MMSE and MoCA were defined in order to analyze whether certain components might more heavily contribute to distinguishing MCI from the HC group for each test. Three domains had items that reflected traditional neuropsychological constructs (orientation, attention and calculation, and comprehension/executive function). The fourth domain, termed “core” was comprised of items assessing three classic symptoms of AD − naming (anomia), new learning (amnesia) and visuospatial ability (agnosia) − reflecting temporoparietal dysfunction that clinicians can easily assess in the office. Items that were not similar between the two tests were excluded from these domains. Items and domains for the MMSE and MoCA are provided in Table 1.

**Table 1:** Comparison of similar items between MMSE and MoCA

Domain names	MMSE		MoCA	
	Items	Points	Items	Points
Orientation	Orientation to time and place	10	Orientation to time and place	6
Attention and Calculation	Spell WORLD backwards^a^	5	Serial 7’s	6
			Vigilance test for letter “A”	
			Digit span (5 forward, 3 backward)	
Comprehension/Executive Function	Follow 3-stage command	4	Verbal fluency for letter F	4
			Trailmaking Test (brief version)	
			Abstraction (word similarities)	
	Read and obey command			
Core	Naming (pencil, watch)	6	Naming (lion, rhinoceros, camel)	12
	Recall (>10 s delay, 3 words),			
			Recall (5 words after 5 min delay)	
	Visuoconstructional (copy intersecting pentagons)			
			Visuoconstructional (copy cube and draw clock face)	
Items not included in domains selected for our analysis	Registration (3 trials, 3 words)	5	Repetition (2 longer sentences)	2
	Repetition (1 short sentence)			
	Write a sentence			
Total Score		30		30

Items were selected to comprise domains used in stepwise regression analyses to distinguish MCI from HC subjects

*ADNI* Alzheimer’s Disease Neuroimaging Initiative, *HC* healthy control, *MCI* mild cognitive impairment, *MMSE* Mini-mental State Exam, *MoCA* Montreal Cognitive Assessment

^a^Serial 7’s were not used for Attention and calculation in the MMSE in ADNI

#### Statistical analyses

Demographic characteristics, MMSE, MoCA, and FAQ scores for each diagnostic group are described using means and standard deviations or frequencies where appropriate. Scatterplots and Pearson correlations were applied for better understanding of the relationships amid the within-case MoCA and MMSE scores for MCI compared to HCs and AD dementia cases. A cutoff of ≥24 points for the MMSE and cutoffs of ≥17, 19, and 23 for the MoCA were denoted in scatterplots to show the lower ends of the potential MCI score ranges.

We prespecified four domains (orientation, attention and calculation, comprehension/executive function, and core) for each test (see Table 1). Stepwise variable selection in logistic regression was then performed to identify domains that most contributed to differentiating MCI from HC for each test.

The equi-percentile equating method with log-linear smoothing [25] was performed on the MoCA and MMSE to develop a score conversion table between these scales. The analysis was performed using the “equate” library in the R statistical program. Receiver Operating Characteristic (ROC) analysis for MCI versus AD dementia was performed to obtain cutoff values with sensitivity and specificity for MoCA scores. Youden indices are reported where the highest value reflects balanced sensitivity and specificity.

All analyses were carried out in SAS version 9.2 unless specified otherwise.

## Results

Table 2 presents demographic, test, and rating scale scores for each diagnostic group. All groups were highly educated. There were significant differences between group means for MMSE, MoCA, and FAQ scores with a greater degree of impairment in the direction as expected (dementia > MCI > HC).

**Table 2:** Demographic, cognitive test and scale scores by diagnostic group

Variable	HC (*n* = 219)	MCI (*n* = 299)	AD dementia (*n* = 100)	*p*-value*
Age (years)	77.69 ± 6.24 (63–94)	74.19 ± 7.91 (56–92)	77.58 ± 7.62 (56–92)	<.0001
Sex (% male)	50.68	60.87	67.00	.0103
Education (years)	16.42 ± 2.72 (6–20)	16.18 ± 2.76 (8–20)	15.75 ± 3.08 (3–20)	.1353
MMSE	29.07 ± 1.24 (23–30)	27.83 ± 1.92 (21–30)	20.31 ± 4.70 (7–30)	<.0001
% subjects with MMSE ≥24	99.54	98.33	21.00	<.0001
MoCA	25.57 ± 2.75 (16–30)	23.41 ± 3.38 (13–30)	15.30 ± 5.51 (1–28)	<.0001
% subjects with MoCA ≥17	99.54	96.32	48.00	<.0001
% subjects with MoCA ≥19	99.09	90.97	36.00	<.0001
% subjects with MoCA ≥23	87.67	65.89	7.00	<.0001
FAQ	0.30 ± 1.06 (0–10)	3.01 ± 4.20 (0–25)	18.63 ± 6.94 (4–30)	<.001
% subjects with abnormal FAQ^a^	0 %	1.7 %	60 %	<.001

Data are expressed as mean ± SD (range) except where frequencies are used for categorical data

*AD* Alzheimer’s disease, *ANOVA* analysis of variance, *HC* healthy control, *FAQ* Functional Activities Questionnaire, *MCI* mild cognitive impairment, *MMSE* Mini Mental State Exam, *MoCA* Montreal Cognitive Assessment, *SD* standard deviation

*chi-square test for categorical variables; F test (ANOVA) for continuous variables

^a^abnormal FAQ defined as at least 3 items each with a score of 3 points

### Test score distributions

**Fig. 1:**
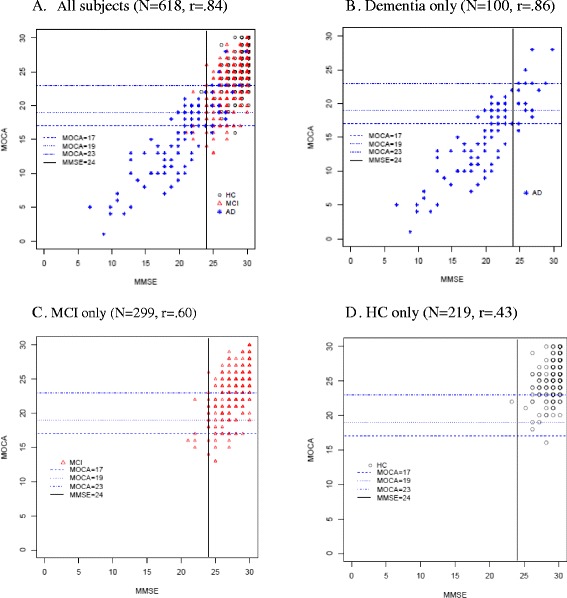
Scatterplots for MMSE and MoCA scores shown by diagnostic group. Graphs are for all subjects (**a**), dementia only (**b**), MCI only (**c**) and HC only (**d**). Pearson correlation coefficients between MMSE and MoCA scores are shown for each graph. Vertical lines denote MMSE standard cutoff of 24 points and horizontal lines denote different proposed MoCA cutoffs for MCI (17, 19 and 23). Note that symbols may represent more than one case at that score

Figure 1 scatterplots show the score distribution relationships between MMSE and MoCA, coded by diagnostic group and with lines denoting various possible cutoff scores. The correlation coefficients between tests were high for all subjects (0.84) and AD dementia (0.86), but lower for MCI (0.60) and HC (0.43).

MCI scores were mostly above the cutoff values, where the lowest MoCA cutoff of ≥17 captured more MCI cases (96.3 %) than did the higher cutoffs; ≥23 missed a number of MCI cases, though it also captured the least number of dementia cases. In contrast to the MoCA, 98.3 % of MCI subjects scored ≥24 on the MMSE, which is within its published normal range. To evaluate the known ceiling effect of the MMSE, we also compared a cutoff of ≥28 on both tests where many more HC and MCI cases had scores of 28 to 30 on the MMSE (71.4 %) than on the MoCA (18.1 %).

Scores for AD dementia cases were distributed widely on both tests, including above and below cutoff lines, revealing a broad range of cognitive impairment severity despite having a dementia diagnosis.

On each test, HC scores were more tightly distributed in the upper right quadrants and overlapped greatly with MCI scores. Nearly all of the HCs scored in the upper right quadrant with 99.5 % above the MoCA cutoff of ≥17 and 99.5 % above the MMSE ≥24 cutoff.

### Strategies to exclude cases overlapping in the MCI range

#### FAQ

Mean FAQ scores were significantly different (*p* < .001) among diagnostic groups where only the AD dementia group was in the abnormal range (see Table 2). Abnormal FAQs were highest in the dementia group (60/100), while MCI had 5/296 and HC had none. MCI cases with abnormal versus normal FAQs, respectively, had mean scores of 19.6 ± 3.7 and 23.5 ± 3.4 on the MoCA, and 27 ± 2.2 and 27.8 ± 1.9 on the MMSE.

#### Domain analysis of MoCA and MMSE

Using stepwise logistic regression to select from among the four prespecified domains, the core domain on both MMSE and MoCA (*p* < .0001) followed by the orientation domain (MMSE, *p* < .0001 and MoCA, *p* = .0009) were selected as contributing significantly to discerning MCI versus HC. The comprehension/executive function and attention and calculation domains were not selected for either test.

### Translation between MoCA and MMSE scores

**Fig. 2:**
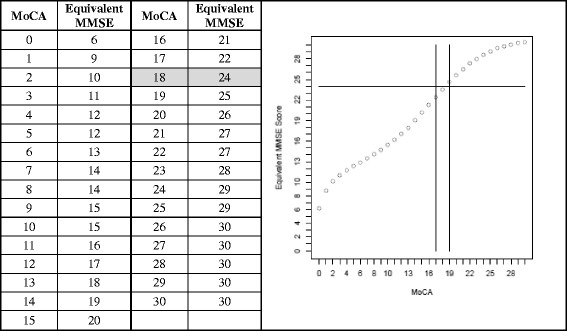
MoCA mapped to MMSE scores using equi-percentile equating method with log-linear smoothing in 618 subjects. Lines in graphs indicate MMSE cutoff of 24 and MoCA cutoffs of 17 and 19. The MoCA value equivalent to a MMSE cutoff of 24 is shaded

Figure 2 describes the equivalent scores when translating between the MoCA and MMSE tests as analyzed in our cohort of all subjects using the equi-percentile method, including a graphic display. A MoCA score of 18 corresponded to an MMSE of 24. MMSE score ranges from 28 to 30 converted to 23–30, and from 22 to 27 to 17–22 on the MoCA. The lowest range on the MoCA (0–16), where many dementia cases predominated, was represented by scores of 6–21 on the MMSE, though the small number of subjects in that lowest score range makes interpretation less confident.

**Fig. 3:**
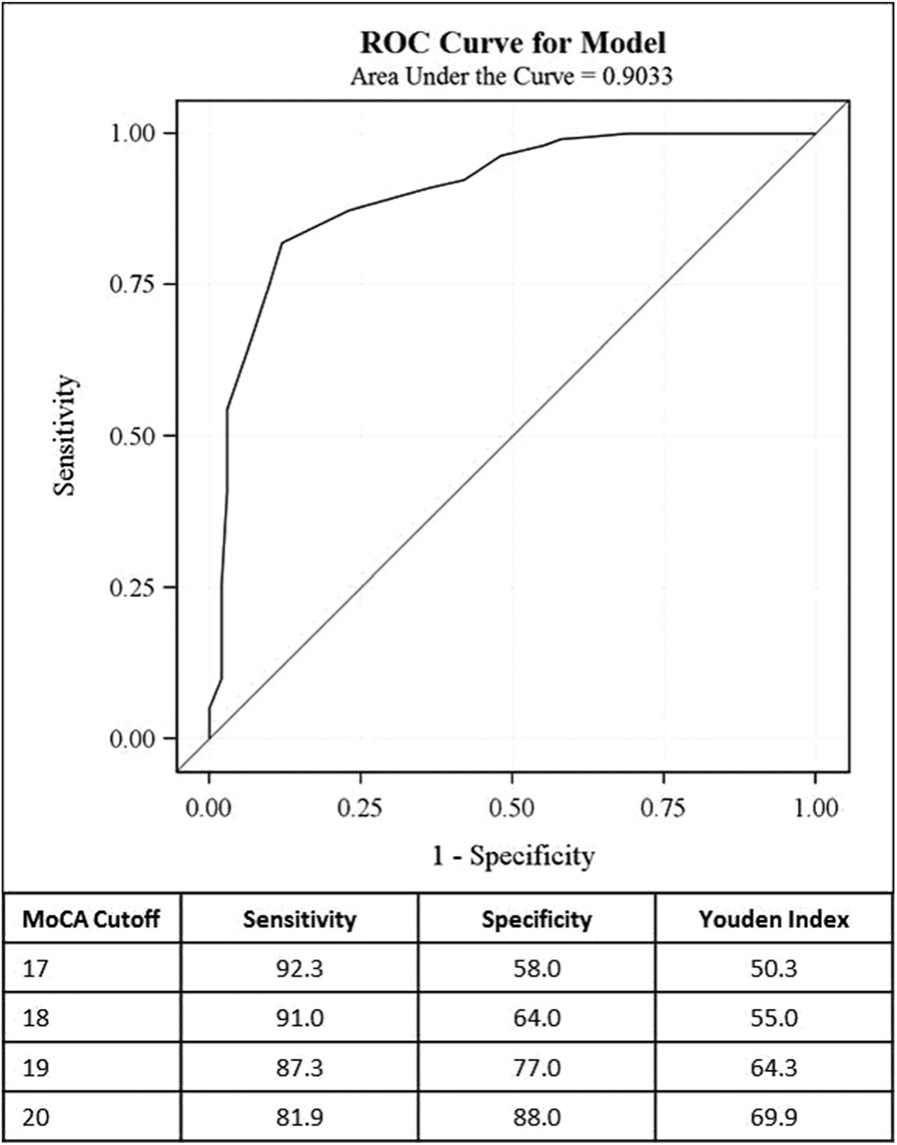
ROC analysis graph of MoCA scores for distinguishing MCI subjects (*n* = 299) from the AD dementia group (*n* = 100) and table for MoCA values to consider as lower cutoff values for MCI (17–20) depending on sensitivity and specificity levels preferred in a given situation

ROC analysis of MoCA scores performed for MCI, as differentiated from AD dementia, had an AUC of 0.9033 (see Fig. 3). A MoCA cutoff score of 17 emphasized sensitivity (92.3 %) with specificity of 58 %, while a cutoff of 19 had somewhat lower sensitivity (87.3 %) and higher specificity (77 %). The Youden index was highest (69.9) for a MoCA cutoff of 20 with the balance of sensitivity (81.9 %) and specificity (88.0 %).

## Discussion

There is growing interest in using the MoCA as an office-based multi-domain cognitive test in lieu of the MMSE due to its apparent advantages for assessing cognitively impaired patients in earlier stages, in part attributed to its greater difficulty and inclusion of executive function items. Our study purpose was to determine MoCA scores that captured the range of MCI severity for clinicians and researchers whereas prior work emphasized distinguishing MCI and dementia from HCs. We found a broader score distribution for MCI subjects on the MoCA than the MMSE, with a reduced ceiling effect. This ceiling effect was also reflected by mean test scores in MCI cases with and without abnormal FAQs that were almost the same on the MMSE but more disparate on the MoCA. The broader MCI score range on the MoCA could be useful for earlier detection when deficits are more subtle and for monitoring cognitive function over time. We anticipated overlap of MCI with HCs at the upper range and with dementia cases at the lower MoCA score range. We realized that applying another strategy would then be required to separate out the overlapping HC or dementia cases, similar to the approach taken by Roalf et al. though they used classification [14]. Because much literature still benchmarks AD cohorts using the MMSE, the way in which MMSE and MoCA scores translate is important; therefore we also report equi-percentile conversion scores. We also performed ROC analysis to help determine the lower MCI cutoff values.

First, we evaluated score distributions and their overlap for the MoCA as compared to the MMSE using ADNI data for 618 cases. The MoCA cutoff of ≥17 points detected most of the MCI cases (96.3 %), similar to the MMSE using a cutoff of ≥24 (98.3 %). Using the equi-percentile approach, a MoCA score of 18 was comparable to MMSE of 24. Using ROC analysis for MCI versus AD dementia, MoCA cutoff values of ≥17 to 19 were chosen to relatively emphasize sensitivity (92.3 to 87.3 %) over specificity (58 to 77 %). The Youden index would suggest a MoCA cutoff of 20 if balancing both (81.9 %, 88.0 %) is desired, which was not the purpose for this study that sought to capture as many MCI cases as reasonably possible. We recommend a lower cutoff of ≥17 and upper cutoff of ≤30 points on the MoCA to capture the range of severity of MCI patients. However, a lower cutoff of ≥18 or 19 could be used, depending on the level of sensitivity and specificity that is desired. Therefore, there is a tight range of MoCA scores (17–19) that could constitute the lower cutoff for MCI based on the different ways we examined these ADNI data.

Many dementia cases were below the cutoffs for MCI on both scales (<24 MMSE; <17 MoCA); however, 48 % of the dementia cases were also captured when using the ≥17 MoCA MCI range. This suggests either the level of cognitive impairment in mild dementia overlaps with that of more severe MCI or some inconsistency for diagnosis when overall clinical judgment using a variety of inputs is done as in ADNI. Irrespective of the reason, we recommend that the MoCA be used in conjunction with a functional measure to help separate the dementia from MCI cases. Using the published FAQ scoring method of 3 points on at least 3 items [24] to denote functional impairment, the dementia groups’ scores were predominantly abnormal whereas most of the MCI and all of the HCs were normal, and the FAQ is easily administered in a clinical setting.

As expected, the HC score range overlapped with MCI on both tests, thus the clinician might wish to perform more sensitive neuropsychological tests or refer such patients to a neuropsychologist to further evaluate whether they are healthy or showing early, subtle deficits. Another approach to help differentiate MCI from HC is to use performance on the core and orientation domains of the MoCA and MMSE, based on our stepwise regression. Executive function was measured on the MoCA but did not distinguish these groups though the core domain which included recall, naming and visuoconstructional ability did. Though it would be easy to get domain subscores in clinical practice, further validation work should be done including determining cutoff values.

Consistent with a greater ceiling effect on the MMSE than MoCA, correlations between tests were lower in the MCI (0.60) and HC (0.43) groups. However, because test distributions were similar at the recommended cutoffs and highly correlated in the dementia group (*r* = .86), these tests can be considered more comparable for use in dementia patients.

Our finding potential advantages of the MoCA over the MMSE in MCI is consistent with a previous report about the MoCA’s high discriminant potential for MCI that was significantly different from that for the MMSE (*p* = .0007), with an area under the curve (AUC) for the MoCA of 0.86 compared to 0.75 for the MMSE [13]. By contrast, they found no difference between the MoCA and MMSE for AD dementia (*p* = 0.1018) with an AUC for the MoCA of 0.98 compared to the MMSE AUC of 0.96 [13], consistent with our finding of a higher correlation between the two scales in the dementia group.

Finally, we used the same equi-percentile equating method noted in Roalf et al. [14] and in similar diagnostic groups. They translated scores between the MMSE and MoCA in a geriatric clinic population that was somewhat younger and less educated than our cohort. Our findings are remarkably similar to theirs across the MCI range, with the same MMSE conversion scores for MoCA 23 and 28, and one point different for MoCA 17 (22 vs 23). Recently, the Roalf equivalency findings were applied to a racially diverse sample to convert MoCA to MMSE scores and found to have high correlation (intraclass correlation coefficient = 0.85, *p* < .001) when compared to actual MMSE scores [26]. Thus these conversions seem to have some consistency across populations. However, our graphic representation revealed an almost linear relationship except at the extreme of scores, probably because the MMSE is less difficult than the MoCA, allowing more impaired patients to score a bit higher at the lower end of the scale, while the least impaired scored higher at the upper end (ceiling effect). Therefore, some caution is advised when interpreting the low end where very few of our cases were severely demented, and our conversion was most different from Roalf et al. [14] in the severe dementia range.

Limitations include our cross-sectional analyses using ADNI data, which may affect the generalizability. ADNI is not necessarily reflective of the general population and has a higher proportion of high cognitive reserve cases, which is protective of onset of AD symptoms [27]. It may be that the MoCA would detect MCI even better in the general population, but this needs to be studied. By ADNI protocol design, if both the MMSE and MoCA tests were administered on the same day, the MMSE was administered first which could contribute to an order effect that altered MoCA scores through either a learning effect for similar items or mental fatigue, especially in demented patients. However, intervening tests were administered to reduce a possible learning effect on the few items that were the same. Since the MoCA is a more difficult test than the MMSE, detection of cognitive impairment may be even better when the MoCA is used alone than what is reported here if an ordering effect had occurred. It should also be noted that these MCI subjects had a memory complaint making the results relevant only to the amnestic MCI subtype. We used the diagnosis associated with our study visit, and not the ADNI entry visit diagnosis when the MMSE inclusion score criteria would otherwise have constrained our MoCA analysis. Elapsed time since entry (at least 6 months) would reduce possible MMSE influence on later diagnosis.

In summary, we contribute uniquely to the growing literature on the use of the MoCA for detection of MCI and its comparison to the MMSE. Based on subjects in ADNI, we recommend a MoCA score range for MCI using a lower threshold of ≥17 to ≥19, depending on the levels of sensitivity and specificity preferred, and an upper limit of 30. Use of a functional tool such as the FAQ can help discern dementia patients with MoCA scores overlapping in the MCI range and more sensitive neuropsychological testing can be done for HCs with overlapping scores. Though our equi-percentile scores were highly comparable to those recently reported, which adds to some confidence to our findings, replication in community populations would strengthen our recommendations.

## Conclusions

We found MMSE scores had a more pronounced ceiling effect than MoCA for HC and MCI cases. Using ADNI data, in order to detect a similar number of MCI cases using an MMSE cutoff of ≥24, the MoCA cutoff needs to be lowered to between ≥17 to ≥19 depending on the preference for more sensitivity or specificity versus dementia. The more difficult content in the MoCA may enhance its sensitivity to detect earlier symptoms when the upper MCI score is 30. We recommend that the MoCA be used in conjunction with a functional scale such as the FAQ to distinguish dementia cases whose scores overlap in the MCI range and a more sophisticated executive function or episodic memory test to distinguish milder MCI as it transitions from normal. Also, based on stepwise regression, testing the orientation or core domain on either the MMSE or MoCA may help distinguish HC from MCI cases, though this needs to be further evaluated in other samples.

## Additional files

Additional file 1:
**List of IRB committees.** (DOCX 30 kb)

## Abbreviations

AD: Alzheimer’s disease
ADNI: Alzheimer’s Disease Neuroimaging Initiative
AUC: Area under the curve
CDR: Clinical Dementia Rating Scale
CI: Confidence interval
FAQ: Functional Activities Questionnaire
HC: Healthy controls
IADLs: Instrumental activities of daily living
MCI: Mild cognitive impairment
MMSE: Mini-mental State Exam
MoCA: Montreal Cognitive Assessment
r: Correlation coefficient
WMS: Wechsler Memory Scale
