# Supplementary material for: Relationship between the Montreal Cognitive Assessment and Mini-mental State Examination for assessment of mild cognitive impairment in older adults
Source: BMC Geriatr. 2015 Sep 7;15:107. doi: 10.1186/s12877-015-0103-3 (PMC4562190; doi:10.1186/s12877-015-0103-3)
Supplement: Additional file 1: — List of IRB committees. (DOCX 30 kb) [file 12877_2015_103_MOESM1_ESM.docx]

| **Site Number** | **Site Name** | **IRB Name** |
| --- | --- | --- |
| 2 | Oregon Health and Science University | Oregon Health and Science University Institutional Review Board |
| 3 | University of Southern California | University of Southern California Health Science Campus Institutional Review Board |
| 5 | University of California, San Diego | UCSD Human Research Protections Program |
| 6 | University of Michigan | University of Michigan Medical School Institutional Review Board (IRBMED) |
| 7 | Mayo Clinic, Rochester | Mayo Clinic Institutional Review Board |
| 9 | Baylor College of Medicine | Institutional Review Board of Baylor College of Medicine |
| 10 | Columbia University | Columbia University Institutional Review Board |
| 11 | Washington University, St. Louis | Office of the Washington University School of Medicine IRB (OWUMC IRB) |
| 12 | University of Alabama at Birmingham | University of Alabama at Birmingham Institutional Review Board |
| 13 | Mount Sinai School of Medicine | Institutional Review Board of the Mount Sinai School of Medicine |
| 14 | Rush University Medical Center | Rush University Medical Center Institutional Review Board |
| 16 | Wien Center | Institutional Review Board of Mount Sinai School of Medicine |
| 18 | Johns Hopkins University | Johns Hopkins University School of Medicine Institutional Review Boards |
| 19 | University of South Florida Health Byrd Alzheimer's Institute | University of South Florida Division of Research Integrity & Compliance |
| 20 | New York University | New York University Langone Medical Center School of Medicine Institutional Review Board Human Research Program |
| 21 | Duke University Medical Center | Duke University Health System Institutional Review Board |
| 22 | University of Pennsylvania | University of Pennsylvania Institutional Review Board |
| 23 | University of Kentucky | University of Kentucky Office of Research Integrity |
| 24 | University of Pittsburgh | University of Pittsburgh Institutional Review Board |
| 27 | University of Rochester Medical Center | University of Rochester Research Subjects Review Board (RSRB) |
| 29 | University of California, Irvine | University of California Irvine Office Of Research Institutional Review Board (IRB) |
| 31 | University of Texas SWMC | The University of Texas Southwestern Medical Center Institutional Review Board |
| 32 | Emory University | Emory University Institutional Review Board |
| 33 | University of Kansas | Human Subjects Committee, University of Kansas Medical Center |
| 35 | University of California, Los Angeles | UCLA Office of the Human Research Protection Program Institutional Review Board |
| 36 | Mayo Clinic, Jacksonville | Mayo Clinic Institutional Review Board |
| 37 | Indiana University | Indiana University Institutional Review Board, Research Compliance Administration |
| 41 | Yale University School of Medicine | Human Investigation Committee Yale University School of Medicine |
| 51 | Jewish General Hospital / McGill U | Research Ethics Board Jewish General Hospital |
| 52 | Sunnybrook Health Sciences | Research Ethics Board Sunnybrook Health Sciences Centre |
| 53 | University of British Columbia | University of British Columbia Clinical Research Ethics Board (CREB) |
| 57 | Saint Joseph's Hospital | University of Western Ontario Research Ethics Board for Health Sciences Research Involving Human Subjects (HSREB) |
| 67 | Northwestern University | Northwestern University Institutional Review Board Office |
| 70 | Nathan Kline Institute for Psychiatric Research | Nathan Kline Institute Rockland Psychiatric Center Institutional Review Board (NKI RPC IRB) |
| 72 | Premiere Research Institute | Western Institutional Review Board |
| 73 | University of California, San Francisco | University of California San Francisco Committee on Human Research (CHR) |
| 82 | Georgetown University | Georgetown University Institutional Review Board |
| 94 | Brigham and Women's Hospital | Partners Human Research Committee |
| 98 | Stanford University | Stanford University, Administrative Panel on Human Subjects in Medical Research |
| 99 | Banner Sun Health Research Institute | Western Institutional Review Board |
| 100 | Boston University School of Medicine | Boston University Medical Campus Institutional Review Board (BU IRB) |
| 109 | Howard University | Western Institutional Review Board |
| 114 | Case Western Reserve University | University Hospitals Case Medical Center Institutional Review Board |
| 116 | University of California, Davis | University of California Davis Office of Research IRB Administration |
| 123 | Dent Neurologic Institute | Western Institutional Review Board |
| 126 | Parkwood Hospital | Western University Health Sciences Research Ethics Board |
| 127 | University of Wisconsin | University of Wisconsin Health Sciences Institutional Review Board |
| 128 | University of California, Irvine - BIC | University of California Irvine Office Of Research Institutional Review Board (IRB) |
| 129 | Banner Alzheimer's Institute | Western Institutional Review Board |
| 130 | Ohio State University | The Ohio State University Institutional Review Board |
| 131 | Albany Medical College | Albany Medical College Institutional Review Board |
| 135 | University of Iowa | University of Iowa Institutional Review Board |
| 136 | Dartmouth-Hitchcock Medical Center | Dartmouth-Hitchcock Medical Center Committee for the Protection of Human Subjects |
| 137 | Wake Forest University Health Sciences | Wake Forest University Institutional Review Board |
| 141 | Rhode Island Hospital | Lifespan - Rhode Island Hospital Institutional Review Board |
| 150 | Cornell Medical Center | Weill Cornell Medical College Institutional Review Board |
| 153 | Cleveland Clinic Lou Ruvo Center for Brain Health (CCLRBC) | Cleveland Clinic Institutional Review Board |
| 168 | Roper St. Francis Hospital | Roper St. Francis Institutional Review Board |
| 941 | Butler Hospital Memory & Aging Program | Butler Hospital Institutional Review Board |
